# Ten-color, whole blood flow cytometric analysis of human myeloid subsets; implications for immune monitoring in cancer patients

**DOI:** 10.1186/2051-1426-1-S1-P102

**Published:** 2013-11-07

**Authors:** Michael P Gustafson, Yi Lin, Mary L Maas, Dennis A Gastineau, Allan B Dietz

**Affiliations:** 1 Laboratory Medicine and Pathology, Mayo Clinic, Rochester, MN, USA; 2 Hematology, Mayo Clinic, Rochester, MN, USA

## 

Myeloid cells comprise a heterogeneous population of cells that include granulocytes and monocytes. Recent data reveals a complex role of myeloid cells as mediators of tumor progression and tumor induced immunosuppression. Myeloid-derived suppressor cells (MDSCs) have been extensively studied in mouse models and are thought to be actively recruited to the tumor and suppress immunity through multiple mechanisms. Characterizations of human MDSCs have been much more problematic due to the lack of equivalent surface markers in mice and the variation and inconsistent use of human cell surface markers. The use of these populations as biomarkers for immunity requires standardization in sample prep and reporting. Typically, blood is prepared for analysis by collection of the mononuclear cell fraction. However, we found that this method can alter the cell populations of interest. For example, paired samples from healthy ten volunteers showed an increase of CD16 expression on monocytes purified by density gradient centrifugation versus those stained directly from whole blood. In addition, whole blood flow cytometry allows universal reporting terms as results can be reported in cells/μL. In effort to begin to standardization human myeloid subsets, we developed a ten color flow panel to analyze unfractionated peripheral blood. As part of this development, we felt it was very important to use surface markers and non-overlapping gating strategies that would enable us to define distinct myeloid populations. We used a combination of ten markers that allows us to measure over 15 myeloid phenotypes including granulocytes (using CD15, CD16 and CD66b), monocyte sub-populations (identified by CD14 and CD16), suppressive monocytes (CD14+HLA-DRlo/neg), myeloid derived suppressor cell populations (LINnegCD33+HLA-DR+), and circulating dendritic cell populations (LINnegHLA-DR+ subgrouped by CD11c and CD123). In addition to typical bivariate plotting strategies, we also used radar plot analyses to look at multiple markers simultaneously. Data from healthy volunteer controls and cancer patients of the non-overlapping myeloid populations will be shown. This approach will allow standardization of the collection and reporting of these critically important populations.

